# Establishment of a Newborn Lamb Gut-Loop Model to Evaluate New Methods of Enteric Disease Control and Reduce Experimental Animal Use

**DOI:** 10.3390/vetsci8090170

**Published:** 2021-08-24

**Authors:** Ambre Baillou, Nathalie Kasal-Hoc, Céline Barc, Juliette Cognié, Anne Pinard, Jérémy Pezant, Julie Schulthess, Pauline Peltier-Pain, Sonia Lacroix-Lamandé, Fabrice Laurent

**Affiliations:** 1 UMR1282 Infectiologie et Santé Publique, INRAE Centre Val de Loire, Université François Rabelais de Tours, 37380 Nouzilly, France; ambre.baillou@inrae.fr; 2 Phileo by Lesaffre, 137 rue Gabriel Péri, 59700 Marcq-en-Barœul, France; j.schulthess@phileo.lesaffre.com (J.S.); p.peltierpain@phileo.lesaffre.com (P.P.-P.); 3 UE1277 Plateforme d’Infectiologie Expérimentale (PFIE), INRAE Centre Val de Loire, 37380 Nouzilly, France; nathalie.kasal-hoc@inrae.fr (N.K.-H.); celine.barc@inrae.fr (C.B.); anne.pinard@inrae.fr (A.P.); jeremy.pezant@inrae.fr (J.P.); 4 UMR85 Physiologie de la reproduction et des Comportements, INRAE Centre Val de Loire, CNRS, IFCE, Université François Rabelais de Tours, 37380 Nouzilly, France; juliette.cognie@inrae.fr

**Keywords:** newborn lamb, multiple intestinal loop model, 3R, *Cryptosporidium parvum*, yeast cell wall fractions

## Abstract

Enteric infectious diseases are not all well controlled, which leads to animal suffering and sometimes death in the most severe cases, in addition to economic losses for farmers. Typical symptoms of enteric infections include watery diarrhea, stomach cramps or pain, dehydration, nausea, vomiting, fever and weight loss. Evaluation of new control methods against enteric infections requires the use of many animals. We aimed to develop a new method for an initial in vivo screen of promising compounds against neonatal diseases such as cryptosporidiosis while limiting experimental animal use. We therefore adapted an in vivo method of multiple consecutive but independent intestinal loops to newborn lambs delivered by cesarean section, in which endotoxin responsiveness is retained. This new method allowed for the screening of natural yeast fractions for their ability to stimulate immune responses and to limit early *Cryptosporidium parvum* development. This model may also be used to investigate host–pathogen interactions and immune responses in a neonatal controlled environment.

## 1. Introduction

Infectious diseases are mainly controlled by the use of vaccines and antimicrobials. Despite major discoveries, some infectious diseases remained refractory to vaccination and treatments and therefore require specific attention together with the new infectious zoonotic threats that increasingly emerge these last decades. The evaluation of new antimicrobials is usually first performed by bioinformatics and/or by in vitro approaches sometimes resulting from High-Throughput Screening (HTS) on miniaturized 2D cell-based assays, evaluating 100,000 or more samples per day [1,2]. Many of these candidates failed when entering clinical trials and have required the unnecessary use of numerous experimental animals. Ex vivo cultures of multipotent or pluripotent stem cells in a three-dimensional (3D) matrix represent major improvements and contribute to reducing animal use [3,4]. However, despite their obvious advantages, these systems cannot yet fully reproduce all the complex characteristics and interactions encountered in vivo, such as the (I) multiplicity and spatial organization of cell types, (II) the immune cell recruitments, (III) the ability to experiment in presence of a full cultivable and non-cultivable microbiota or (IV) to reproduce the peculiarity of a specific age of development e.g., neonatal period, etc. In addition, these models are usually unsuitable for assessing the mechanism of physiopathology resulting from infectious processes. Until technical progress can resolve these limitations, it is therefore important to develop or improve in vivo methods to minimize the use of animals and take full advantage of each experiment in accordance with the 3R rules (Replacement, Reduction and Refinement) [5].

Neonatal enteric infections result essentially from the infection of bacterial, parasitic, or viral pathogens and they have profound effects on intestinal absorption, nutrition, and youth development as well as on global mortality. Cryptosporidiosis due to the zoonotic protozoan parasite *Cryptosporidium parvum* (*C. parvum*) is characterized by infection of the epithelial cells of the small intestine, mainly in the ileum, leading to acute diarrhea and dehydration that may lead to death in severe cases [6]. This disease represents a true one-health threat with severe consequences for human and animal health [7,8]. Extensive new epidemiology studies revealed cryptosporidiosis to be the second leading cause of death in children due to diarrheal disease worldwide [9], and is the first cause of diarrheal enteric disease in young ruminants in France [10]. There is no vaccine and a very limited chemotherapy available for animals and humans [11]. 

We aim to evaluate natural alternatives for controlling cryptosporidiosis using lambs as a target species and also as a model for larger animals such as calves. Indeed, lambs represent a cost-effective model and harbor similar development of gut lymphoid tissues at birth to other young ruminants. We intent to stimulate the immune responses of animals from birth with colostrum supplemented with natural products such as yeast cell wall (YCW) fractions that contain TLR2, TLR4 and Dectin-1 receptor ligands. However, intestinal immune tolerance initiates rapidly after birth in response to microbial colonization gained during vaginal delivery and subsequently via colostrum, milk and multiple contacts with the environment. This immune tolerance is characterized by a rapid hypo-responsiveness to microbial antigens as demonstrated in mouse models [12,13]. 

We therefore developed an in vivo model suitable to investigate both host–pathogen interactions in a controlled environment and to evaluate new natural antiparasitic compounds. Our model relies on two previously described gut-loop models: one performed with fetal lambs (120 to 130 days of gestation) but with just a single loop [14] and another one made with 4–6 month-old lambs [15]. The surgical procedure was therefore successfully adapted right after birth to cesarean-born lambs and allowed to produce an isolated intestinal segment in the ileum area free of microbiota and immune system stimulation. Multiple loops were created in the isolated intestinal segment which allowed for the evaluation endotoxin responsiveness, immune responses, parasite replication in presence or not of YCW fractions. This new model combines different specificities which were required for our investigations such as (1) a newborn model for neonatal enteric disease study, (2) sterility for evaluating the immunomodulatory properties of natural compounds provided in the first colostrum and (3) a large number of intestinal loops per animal to perform multiple comparisons of selected compounds while severely limiting the use of experimental animals. 

## 2. Materials and Methods

### 2.1. Development of a New Animal Model

#### 2.1.1. Ethic Statements

Animal needs were met in accordance with the European Community Council Directive 2010/63/EU (Decree: 2013-118 01/02/2013). The experimental facilities had received authorization to house experimental animals from the local bureau of veterinary services (Indre-et-Loire, France, authorization N°: D 37-175-3), and all the experimental procedures were approved by the Val de Loire Ethics Committee (authorization N° APAFlS#16870-201809261558973 v2). All animal experimentations have been performed in the Infectiology of Farm, Model and Wildlife Animals Facility (PFIE, Centre INRAE Val de Loire available online: https://doi.org/10.15454/1.5572352821559333E12 (accessed on 23 August 2021); member of the National Infrastructure EMERG’IN). All the personnel involved had special training in animal care, handling and experimentation, as required by the French Ministry of Agriculture.

#### 2.1.2. Neonatal Care of Newborn Lambs after Cesarean Surgery

The pregnant Préalpes-du-Sud ewe selected to give birth to two lambs was subjected to classical cesarean surgery. After stimulation of the respiratory function, right after birth, they were positioned in lateral decubitus and received intranasally one to two drops of doxapram (Dopram^®^, Vetoquinol, Lure, France) to stimulate the respiratory rate. The umbilical cord was disinfected with Vetedine^®^ (Vetoquinol, Lure, France) and newborns were then placed together in close contact in the housing box, in a dry and warm environment, under a heating lamp. After 30 min, one of the two lambs was operated. 

#### 2.1.3. Intestinal Loop Surgery

Preparation: The lamb was placed on a heated neonatal resuscitation table and received isoflurane via a face mask for general anesthesia and buprenorphine (Buprecare^®^ Multidose 0.01 mg/kg, Axience, Pantin, France) for analgesia by intramuscular injection. The lamb was then intubated with an endotracheal tube (internal diameter 4 to 5 mm, RÜSCH^®^, Teleflex, Wayne, NJ, USA) and put on assisted ventilation (CPV mode: controlled pressure ventilation, Fabius^®^ Tiro, Dräger, Lübeck, Germany), with a 50/50 oxygen and air mixture. The monitoring system installed (BLT, M8500) allowed to continuously monitor rectal temperature, heart and respiratory rates, oxygen saturation (SpO_2_) and End Tidal CO_2_ (ETCO_2_). An intravenous catheter was placed in the cephalic vein and a warm infusion of sterile isotonic Ringer-Lactate saline solution (Osalia, Paris, France) was started at a rate of around 10 mL/kg/h via a flow regulator. 

Surgical intervention: The surgical area was disinfected and a subcutaneous injection of 1 mL of lidocaine was performed at the level of the midline. After a few minutes, a 10-cm skin incision was made in the midline, caudal to the umbilicus. After dissection of the subcutaneous tissue and opening of the abdominal cavity, the urachus duct was reclined. Sterile compresses moistened with prewarmed (+37 °C) sterile saline were placed all around the surgical wound. The intestinal tract was exteriorized on the compresses, the area of interest (from the ileocecal fold) was identified and the rest was reintroduced into the abdomen. The externalized portion was regularly humidified with sterile warm saline throughout the procedure.

**Figure 1:**
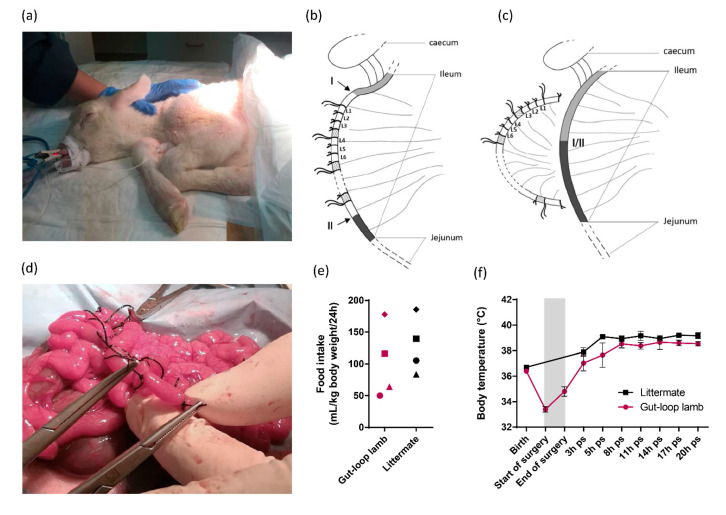
Intestinal loops surgery on the ileum of cesarean-section born lamb. (**a**) Picture of lamb that was anesthetized before surgery, intubated with endotracheal tube and put on assisted ventilation; (**b**) Schematic representation of the intestinal loop generation in the ileum of caesarean-born lamb. Series of triplicates of loops (L1, L2, L3) were generated between the two areas (I and II) indicated by arrows. Interloop region separates series of triplicates and are indicated in shaded areas. The first ligature of the triplicates harbors longer ends, so do the one of each triplicate to facilitate the identification; (**c**) Schematic representation of end-to-end anastomosis is visualized (I/II) allowing intestinal transit restauration and the separation of the intestinal segment containing the isolated loops; (**d**) Picture of a loop creation within intestinal segment during surgery; (**e**) Food intake represented by the volume of milk drunk per ml and per kg of lamb body weight per 24 h for the gut-loop lamb and its littermate. Each point shape corresponds to an independent experimentation (*n* = 4 for each group); (**f**) Body temperature (°C) (mean ± SEM) was monitored from birth to 20-h post-surgery (ps) for the gut-loop lamb and its littermate. The grey box corresponds to the surgery intervention (from start to end).

Creation of loops and inter-loops: A silk ligature (dec. 3, SILK^®^, SMI, St Vith, Belgium) was made approximately at 5 cm from the ileocecal fold (retrograde direction). This 5 cm area is the site of the distal enterotomy. Then, moving up the small intestine, ligatures were placed at approximately 2–3 cm intervals to generate three consecutive loops, and an interloop region which separates each set of triplicates. This can be repeated as many times as necessary before the continuous ileal Peyer’s patch disappears (Figure 1b). Long ends were performed to allow the identification of each triplicate of loops plus the interloop region to be easily clamped for reliable tracking during the loop injection stage. The lumen of the gastrointestinal tract was not rinsed before the ligatures were made, the intestinal contents remained therefore intact. 

Double enterotomy, end-to-end anastomosis: A double enterotomy was performed on each extremity of the area containing loops (Figure 1c). The four ends created were immediately wiped with a sterile compress moistened with warm saline. To avoid leakage or adhesion, each end of the isolated intestinal segment was sutured with a single suture (dec. 1.5, MONOCRYL^®^, Johnson & Johnson, New Brunswick, NJ, USA) and a jejuno–ileal anastomosis was performed to allow normal intestinal transit before gently placing the entire externalized digestive tract back into the abdominal cavity. It should be noted that the vascularization of the intestinal loops after the separation of the ileal segment was maintained. The abdominal cavity was rinsed several times with sterile warm saline. The abdominal wall was stitched with a single suture (dec. 2, SAFIL^®^, B. BRAUN, Melsungen, Germany) followed by subcutaneous and cutaneous sutures and finally, the wound was disinfected. Isoflurane supply was cut off, the animal was put on assisted ventilation with a 100% oxygen mixture and then positioned in a lateral recumbence to accelerate awakening. The animal was regularly “switched” to spontaneous ventilation (Man/Spont) and received an intravenous injection of doxapram (0.87 mg/kg, Dopram-V Injectable^®^, Vetoquinol, Lure, France), repeated once if necessary, to help spontaneous breathing. The animal was then extubated, the catheter was removed and the animal was placed in the housing box with heat lamp, in direct contact with the control lamb from the litter.

Post-operative care: The lambs were fed as the first signs of awakening were observed (Colostromix^®^, Technovet Eurotonic, Briec, France). The volume and number of feedings were adapted upon lamb request. Analgesia was continued with four intramuscular injections per day of buprenorphine (0.01 mg/kg). The criteria for assessing the animal’s clinical condition and possible pain were primarily behavioral: vocalizations (which do not occur after drinking), lordosis, prostration or apathy, absence or too frequent stands up, disinterest in the surroundings, low head carriage or low ears, etc. At every visit to feed them (3 h interval), the surgical wound, rectal temperature, quantity of ingested milk, stool and urine output were controlled. No additional enrichment was provided other than the presence of a littermate from the same litter and all physical contact with the animal technicians, particularly during feedings, which is particularly important at this physiological stage.

Sample recovery: After 24 h, the animal was injected intramuscularly with xylazine (0.5 mg/kg, ROMPUN^®^, Bayer, Leverkusen, Germany) and euthanized intravenously with pentobarbital (180 mg/kg, DOLETHAL^®^, Vetoquinol, Lure, France). The animal was then bled and the small and large intestine was harvested in a whole (duodenum to colon), then samples were taken from each intestinal loop. The unique 24 h-time point was selected to limit animal consumption based on the compromise between assessing the innate immune response to immunostimulants and allowing sufficient time to measure the effect on parasite invasion and development.

### 2.2. Use of the Gut-Loop Model for Investigating Immune Responses and Infection with C. parvum

#### 2.2.1. Intestinal Stimulations

Intestinal responses to immunostimulants were evaluated with isolated loops in lambs and ex vivo with explants. Just after the end-to-end anastomosis, 150 µL of stimulants were injected into loops with an 8-mm 30G needle at the following concentrations: bacterial lipopolysaccharide (LPS) (10 µg/mL, LPS-EB, Invivogen, San Diego, CA, USA), R848 (10 µg/mL, Resiquimod, Invivogen, San Diego, CA, USA) and yeast cell wall fraction 1 (YCW1) or 2 (YCW2) (5 mg/mL, Phileo by Lesaffre, Marcq-en-Barœul, France). The two YCW were obtained from a *Saccharomyces cerevisiae* (*S. cerevisiae*) strain and differed in their polysaccharide composition. YWC1 is composed of an equilibrated content between β-glucans and mannoproteins (at minima 20% of each sugar compound), whereas YCW2 is enriched in β-glucans (50%). Twenty-four hours after surgery, animals were euthanized and intestinal tissues were collected either from the loops or from the ileum reconnected to the intestinal transit, and explants were generated with biopsy punches of 8 mm diameter. Tissue samples were incubated in culture medium (RPMI; 10% FBS; 100 U/mL Penicillin; 100 µg/mL Streptomycin; 100 µg/mL Normocin; 250 ng/mL Amphotericin B) alone or with LPS at a concentration of 10 µg/mL, for 4 h at +37 °C and 5% CO_2_. Three explants per condition were used, referred as triplicates.

#### 2.2.2. RNA Extraction and RT-qPCR 

RNA extractions from explants or pieces of loop were performed after homogenization in TRIzol (Invitrogen, Waltham, MA, USA) with an Ultra-turrax and processed according to manufacturer’s recommendations. Total RNA was reverse transcribed with the iScript RT SuperMix kit (Biorad, Hercules, CA, USA) and amplified by quantitative real-time PCR (RT-qPCR) in presence of EVA^®^ Green using CFX96 Touch Real-Time PCR Detection System (Biorad, Hercules, CA, USA). Results were expressed as 2e^−ΔCt^, following normalization with three stable reference genes (*hprt*, *gapdh* and *actb*). mRNA was quantified for *il1α*, *il1β*, *mx1*, *cxcl1*, *cxcl2*, *cxcl8* and *tnfα* genes known to be upregulated by YCW extracts and/or TLR-agonists used in this study, LPS and R848 (see Table S1 for primer sequences). 

#### 2.2.3. *C. parvum* Infection 

Intestinal loops were injected with 150 µL of physiological serum (0.9% NaCl) or with 150 µL of parasite solution containing 1.5 × 10^4^ or 1.5 × 10^5^ oocysts of *C. parvum* in presence or not of yeast cell wall fractions. The *nluc*-INRAE transgenic strain of *C. parvum* [16], which expresses the nanoluciferase enzyme was used in the study to quantify parasite load. All intestinal samples were collected after 24 h of infection with *C. parvum*.

#### 2.2.4. Parasite Burden

The level of infection in the intestinal loops was determined by two complementary methods: The assessment of the luciferase activity of the *C. parvum nluc*-INRAE transgenic strain and the quantification of the *C. parvum 18S* (*Cp18S*) gene expression by RT-qPCR. For relative light unit (RLU) quantification, 500 µL of lysis buffer (50 mM Tris HCl; 2 mM DTT; 2 mM EDTA; 10% glycerol; 1% Triton) were added to biopsy punch, followed by a 30 min incubation at +4 °C. Samples were then vortexed during 1 min and centrifuged at 12,000× *g* for 1 min. Twenty-five µL of supernatant were mixed with 25 µL of Nano-Glo^®^ substrate (1/50, Promega, Madison, WI, USA) and luminescence activity measured with a GloMax^®^ plate reader (Promega, Madison, WI, USA). The values were expressed in RLU per cm^2^ of intestinal tissue. *Cp18S* gene mRNA was quantified using the forward 5′-TAGAGATTGGAGGTTGTTCCT-3′ and reverse 5′-CTCCACCAACTAAGAACGGCC-3′ primers and results are expressed as 2e^−ΔCt^, after normalization with the expression of three reference genes (*hprt*, *gapdh* and *actb*).

#### 2.2.5. Histology and Immunofluorescence

Following dissection and PBS wash, intestinal samples were immediately incubated in a 4% paraformaldehyde PBS solution during 24 h at +4 °C for tissue fixation. After 2 washes of 4 h at +4 °C in PBS, samples were incubated in a 30% sucrose PBS solution before inclusion in OCT (Cellpath, Newtown, UK) and conserved at −20 °C until histological analyses. Seven µm-thick histological sections were realized with the 3050S Leica cryotome (Leica, Wetzlar, Germany).

Intestinal sections were incubated with a rat polyclonal serum against oocyst antigens (as previously used [17]), overnight in the dark at +4 °C and then washed twice with PBS. The secondary antibody coupled with Alexa 594 (Invitrogen, Waltham, MA, USA) was added for 1 h in the dark and washed twice with PBS. Finally, cell nuclei were stained with Hoechst (Invitrogen, Waltham, USA) during 2 min and then washed twice with PBS. Immunofluorescent staining of *C. parvum* were analyzed by microscopy with the Eclipse 80i Nikon Microscope (Nikon, Tokyo, Japan).

#### 2.2.6. Statistics

Statistical analyses were performed using the GraphPad Prism v6 software (GraphPad Software, San Diego, CA, USA). The Kruskal–Wallis non-parametric test and the Dunn’s multiple comparison test were used to determine the significance of difference for parasite load determination by luminescence measure and RT-qPCR between the different conditions. *p*-values of less than 0.05 were considered statistically significant. The linear regression analysis was used to establish the correlation curve between parasite load evaluated by luminescence measure and *Cp18S* gene expression.

## 3. Results

### 3.1. Establishment of Gut-Loop Model on a Cesarean-Born Neonatal Lamb

In order to be able to compare the immunomodulatory properties of natural compounds in the intestinal response of newborn lambs without prior interference with natural microbial stimulation, we adapted the gut-loop model to cesarean-born lambs (Figure 1a). All physiological parameters were constantly recorded during the surgery (SpO_2_, ETCO_2_, respiratory and heart rates, body temperature) and no loss was registered. The ligatures were made in the ileum of lambs since it is the main infected area in the case of natural infection by *C. parvum*. We performed set of triplicates for each treatment to be used, separated by an interloop region to avoid diffusion of the treatment to another adjacent treatment to be administered (Figure 1b). Leakage between loops were never observed confirming the effective sealing of each loop. At the end of the loop’s generation, an end-to-end anastomosis was performed to reconnect the normal transit that will occur when lambs will first feed, and to allow the separation of the intestinal segment containing the isolated loops (Figure 1c). With the practice and experience gained over the first surgeries, the veterinary surgeon can now make around 25–30 intestinal loops (Figure 1d). The gut-loop lamb animals used to evaluate efficacy of immunostimulatory compounds were maintained with their littermates for 24 h before euthanasia and none presented abnormal behavior. Food intake was slightly lower in gut-loop lambs, but all animals were regularly fed on several occasions during the first 24 h (Figure 1e). In gut-loop animals, the body temperature drops before the beginning of the surgery and then regularly increases to reach similar temperature as littermate (Figure 1f), thanks to the heated neonatal resuscitation table, the heat lamp in the housing box and the early food intake following surgery.

### 3.2. Innate Immune Response to LPS Stimulation of Sterile Gut Loop

**Figure 2:**
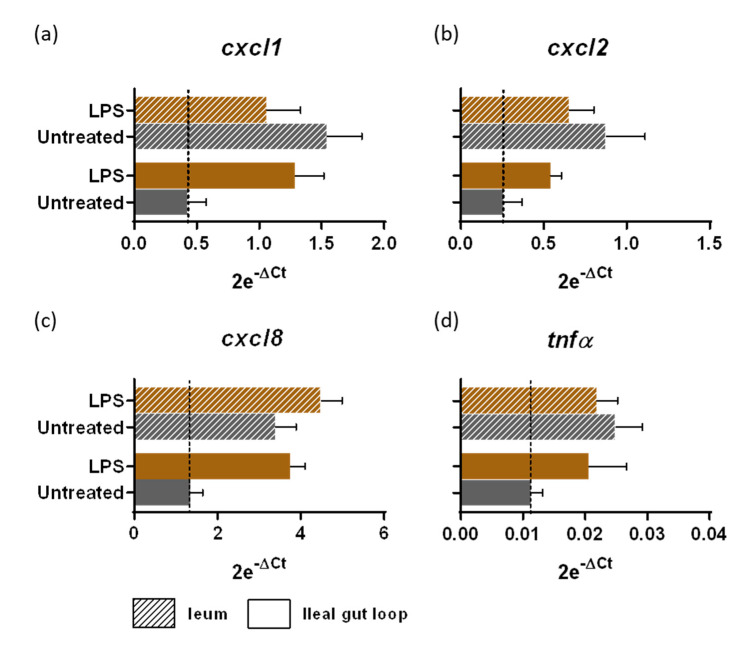
Intestinal responsiveness to LPS stimulation within the gut loop generated with a cesarean-born neonatal lamb. Intestinal tissues from the ileal gut loops and the ileum were recovered from the same neonatal lamb 24 h post-surgery. Small pieces of intestine, designed as “explants”, were placed in a CO_2_ incubator at 37 °C in culture medium alone (untreated) or with LPS at a concentration of 10 µg/mL for 4 h (*n* = 3 explants per condition). Explants were processed for RNA extraction and *cxcl1* (**a**), *cxcl2* (**b**), *cxcl8* (**c**) and *tnfα* (**d**) gene expressions were quantified by RT-qPCR. Results are expressed as 2e^−ΔCt^ (mean ± SEM), following normalization with the expression of three reference genes (*hprt*; *gapdh*; *actb*).

Intestinal immune tolerance initiates within the first hours of life in response to microbial colonization gained during vaginal delivery and subsequently via colostrum, milk and multiple contact with environment. This immune tolerance is characterized by a rapid hypo responsiveness to microbial antigens as demonstrated in mouse models [12,13]. We aim to stimulate the immune responses of animals from birth with colostrum supplemented with natural products and in particular YCW fractions that contain TLR2 and TLR4 ligands. This led us to use lambs born by cesarean-section and whose intestine had not yet been in contact with a microbiota. In order to validate the requirement of using cesarean-born lambs, we assessed LPS responsiveness of isolated ileal loops and of the ileum exposed to microbial colonization 24 h after birth. Chemokines (CXCL1, CXCL2, CXCL8) and TNFα, known to be induced after LPS stimulation, were compared by transcriptomic analysis in both conditions. Our results (Figure 2) demonstrated that in isolated loops LPS induced a chemokine response and TNFα expression while in the connected ileum exposed to microbial and alimentary antigens, these levels were already upregulated and LPS stimulation was without clear additive effect except for a slight increase in cxcl8 mRNA expression. In order to compare the potential of microbial productsm it is therefore necessary to use a cesarean-born lamb that remains fully responsive to microbial ligands. 

### 3.3. Innate Ileal Immune Response to TLR-Ligands and YCW Fractions in Isolated Loops

**Figure 3:**
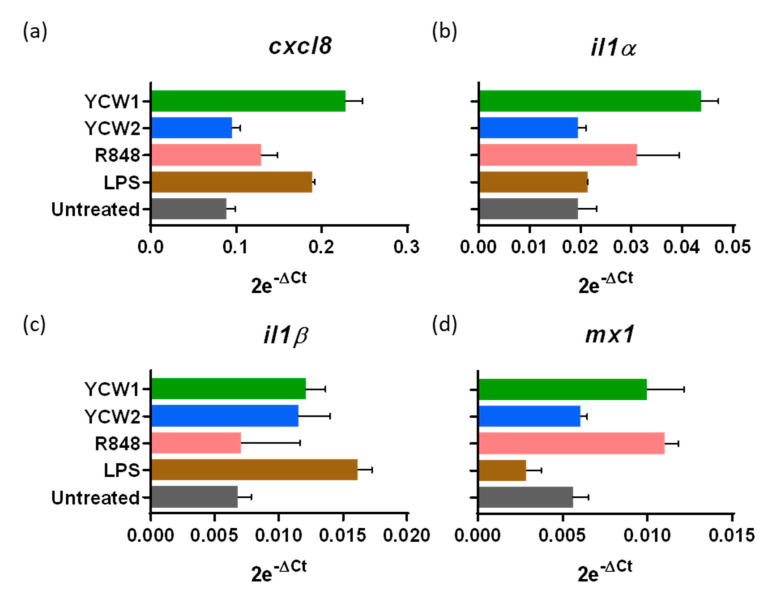
Innate ileal immune response to TLR-ligands and yeast cell wall fractions. Lamb’s gut-loops were stimulated in vivo immediately after the surgery procedure by in situ injection of LPS (10 µg/mL), R848 (10 µg/mL), yeast cell wall fraction 1 (YCW1) or 2 (YCW2) (5 mg/mL) into distinct intestinal loops. Twenty-four hours later, intestinal loops were recovered and samples processed for RNA extraction. *cxcl8* (**a**), *il1α* (**b**), *il1β* (**c**) and *mx1* (**d**) gene expressions were quantified by RT-qPCR. Results are expressed as 2e^−ΔCt^ following normalization with the expression of three reference genes (*gapdh*; *hprt*; *actb*).

YCW principal components are β-glucans and mannoproteins, known to stimulate immune responses through multiple Pattern Recognition Receptors (PRR), mainly TLR2, TLR4 and Dectin-1 [18]. We investigated the innate immune response in ileal loops to two YCW from *S. cerevisiae*, differing in their polysaccharide composition. As controls, we used, *Escherichia coli* LPS, a TLR4-ligand, and R848, a synthetic TLR7-8 viral single-stranded ribonucleic acid mimic-ligand. We compared the responsiveness to these different agonists after 24 h of stimulation. YCW1 induced the mRNA expression of CXCL8 chemokine, IL1α, IL1β proinflammatory cytokines and the interferon-induced Mx1 while β-glucans enriched fraction only induced IL1β (Figure 3). LPS and R848 presented distinct cytokinic responses with R848 inducing the Mx1 upregulation while LPS was more prone to upregulate CXCL8 chemokine. These results confirm the potential of the neonatal gut-loop model to be used as a screening method to evaluate the immunostimulating properties of various compounds using a single animal.

### 3.4. Evaluation of YCW Fractions on Early Cryptosporidium parvum Invasion and Development

**Figure 4:**
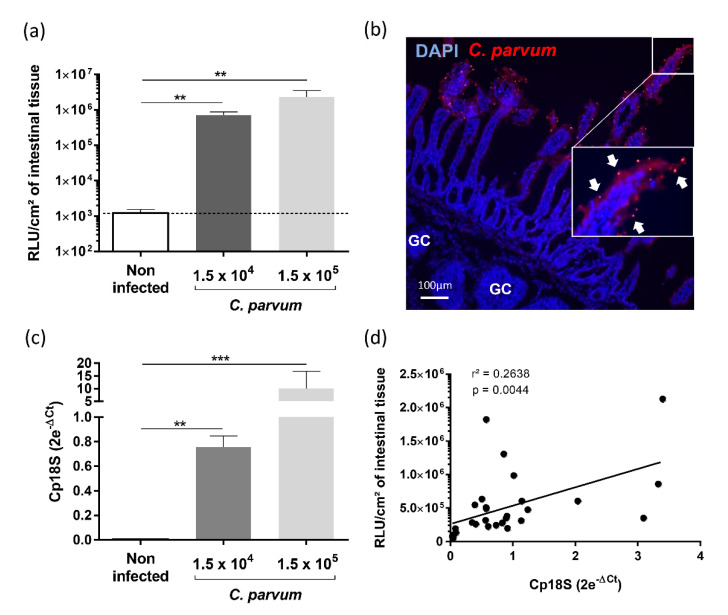
Establishment of *Cryptosporidium parvum* experimental infection in the gut-loop model. Data are cumulative results from three independent experimentations each performed with one newborn lamb. For each experimental condition, three loops were used; (**a**) Parasite load were determined by measuring luminescence activity in the ileal tissue from the loop 24 h after infection with oocysts of *C. parvum nluc*-INRAE transgenic strain (*n* = 9). Results are expressed as RLU per cm^2^ of intestinal tissue (mean ± SEM); (**b**) Immunofluorescence microscopy of ileal tissue section from a *C. parvum* infected gut loop (1.5 × 10^5^ oocysts) 24 h after infection. Parasites were stained in red with anti-*C. parvum* antibodies and nuclei in blue with DAPI. “GC” corresponds to the germinal centers of the ileal Peyer’s patch. White arrows indicate parasites developing into intestinal epithelial cells lining the ileal villi; (**c**) Quantification of *Cp18S* gene expression in the intestinal loops was performed on the same samples as above. RNA was extracted from a piece of each intestinal loop after 24 h of infection with *C. parvum nluc*-INRAE transgenic strain, reverse transcribed and amplified by quantitative PCR. Results are expressed as 2e^−ΔCt^ (mean ± SEM) following normalization with the expression of three reference genes (*gapdh*; *hprt*; *actb*); (**d**) Correlation between parasite load evaluated by luminescence and *Cp18S* gene expression was determined by linear regression analysis. Statistical analyses in (**a**,**b**) were realized with the Kruskal–Wallis non-parametric test and the Dunn’s multiple comparison test, and significative difference was determined by a *p*-value < 0.05 (** *p* < 0.01, *** *p* < 0.001).

To evaluate if immune stimulation with YCW fractions can reduce *C. parvum* infection with a limited number of animals, we used the benefit of the gut-loop model in providing multiple experimental conditions in a single animal. To evaluate *C. parvum* infection and development, we used a previously generated transgenic strain *Cp-Nluc*-INRAE allowing simple and accurate measurement of luciferase activity on tissue samples [16]. Two doses of *C. parvum* oocysts (1.5 × 10^4^, 1.5 × 10^5^) were inoculated in triplicate in independent loops and in both condition, parasite invasion and development were monitored by luciferase activity (Figure 4a). We confirmed the early *C. parvum* development within 24 h of infection by immunofluorescence microscopy, showing parasites developing into intestinal epithelial cells lining the ileal villi (Figure 4b). This result was also confirmed by RT-qPCR on *Cp18S* gene (Figure 4c) on tissue samples recovered from the same ileal loop as for luciferase activity. The correlation between the two variables presented a r^2^ of 0.26 and a *p* value of 0.0044 (Figure 4d). 

**Figure 5:**
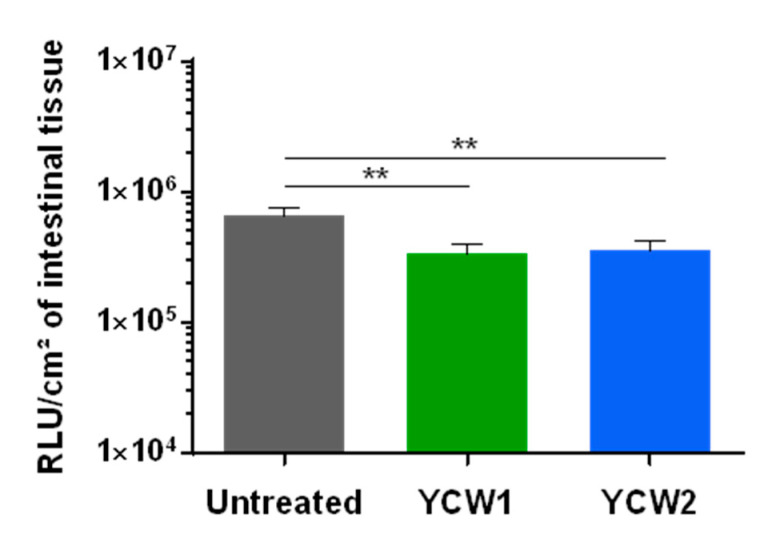
Evaluation of yeast cell wall fractions on early *Cryptosporidium parvum* invasion and development. Data are cumulative results from three independent experimentations each with one newborn lamb. For each experimental condition, three loops were used. Loops were infected by in situ injection of 1.5 × 10^4^ oocysts of *C. parvum nluc-INRAE* transgenic strain alone (untreated), or associated with YCW1 or YCW2 at a concentration of 5 mg/mL. Parasite loads were evaluated by quantifying luminescence in intestinal tissues after 24 h of infection. Results are expressed as RLU per cm^2^ of intestinal tissue with a logarithmic scale (mean ± SEM). Statistical analyses were performed with the Kruskal–Wallis non-parametric test followed by the Dunn’s multiple comparison test (** *p* < 0.01).

The control of cryptosporidiosis is still limited and the search of natural alternative highly anticipated. With the same animal and benefiting from the multiple experimental possible conditions offered by the gut-loop model, we investigated the ability of two YCW fractions to limit *C. parvum* development. Both yeast fractions presented a limited but significant effect on *C. parvum* development (Figure 5), thus validating the gut-loop model to investigate new alternatives of treatment against this disease together with a limited use of experimental animals.

## 4. Discussion

Neonates have an underdeveloped immune system at birth explaining their high susceptibility to infectious diseases. The high incidence of diarrhea in young ruminants must be addressed as soon as possible, as it results in the majority of mortality and morbidity [19,20]. Neonatal diseases, in particular those well controlled by neutralizing antibodies can often be prevented by maternal vaccination with vaccines administered before or during pregnancy. Multivalent vaccines against calf scours currently available are designed to prevent or limit infections with rotavirus, coronavirus, *E. coli* (K99) and *Salmonella* infections. There is no vaccine for *Cryptosporidium* to date which might be explained by the fact that antibodies seem to play a limited role in the protection process [21]. Therefore, alternative control strategies have to be investigated. 

To promote gut health and improve growth, supplements containing immunoglobulins or mineral and vitamin complements, or with probiotics such as *Enterococcus faecium* can be orally given to young ruminants just after birth [22,23,24]. Live yeast and yeast-derived products are also extensively used as probiotic and prebiotic, respectively, for farm animals to reduce the severity of diarrhea by preventing pathogenic bacteria from binding to intestinal epithelial cells or by modulating gut mucosal immunity [25,26,27]. Indeed, YCW fractions are known for their immunostimulatory properties, they harbor ligands for various innate receptor such as TLR2, TLR4 and Dectin-1. They have previously shown to be effective against cryptosporidiosis and pathogenic bacterial colonization in young ruminants. Mammeri et al. identified the anti-cryptosporidial activities of chitosan, a natural polysaccharide present in YCW in in vitro and mouse studies [28]. In another study, yeast culture enriched with mannan-oligosaccharides were fed in milk to Holstein heifer calves enrolled at 4 to 12 h of age, and this led to the presence of fewer *Escherichia coli* and pathogenic *E. coli* compared with control calves [29].

In addition to their immunostimulatory properties, YCW products are suggested to be anti-adhesive agents able to reduce adhesion of intestinal pathogens [30,31]. *Cryptosporidium spp.* are known to express the lectin galactose/*N*-acetyl-D-galactosamine, which facilitates their adhesion to host epithelial cells via interaction with sulfated proteoglycans [32,33]. Therefore, further investigations are required regarding the precise mechanism of inhibition of *C. parvum* observed with YCW extracts while considering the role of polysaccharide composition.

We also wish to investigate if immunostimulatory components administered with the colostrum can strengthen immune system of neonatal ruminants and reduce the incidence of cryptosporidiosis. Comparing multiple derivates from yeast or other sources will require the use of many animals. In order to follow reduce animal consumption, we aimed to develop a gut-loop model adapted to caesarean-born animals. This surgical model, although invasive by definition, fits well with the 3Rs principle. With the practice and experience gained over the first surgeries, we can now make around 25–30 intestinal loops within the ileal Peyer’s patch area which gives the opportunity to produce replicates and/or evaluate many anti-infectious products with just a single newborn animal. With this gut-loop model, the animal is its own control which reduces variability of responses especially in non-inbred animals that vary substantially in their genetic. We took a particular care to provide the best veterinary practices during surgery, anesthesia and analgesia, neonatal nursing, pre- and post-operative care. All of the contact related to the animals were optimized to avoid or limit pain and discomfort. After surgery, the animals fed naturally and did not show any particular behavior compared to its littermate. 

The model is also flexible on the section of the intestine that could be investigated. Indeed, the small intestine contains distinct areas of organized lymphoid tissues at birth like Peyer’s patches that are known to be the major inductive sites of immune responses. Ruminants possess jejunal Peyer’s patches (JPP) that retain classical functions of intestinal Peyer’s patches founds in mouse and human such as antigen sampling through Microfold cells and T- and B-cell activation, but also a peculiarly long ileal Peyer’s Patch (IPP), which extends one meter along the terminal small intestine and which is known to be a primary lymphoid organ of B-cell development [34]. Since *C. parvum* infect primarily the distal small intestine, for our own investigations the intestinal loops were therefore generated in the ileum.

In order to compare various immunostimulant candidates containing TLR ligands we needed to use caesarean-section born lamb to have sterile intestinal environment mimicking the first encounter of the yeast derivates when administered with the first yeast-supplemented colostrum and putative interference with the presence of endotoxins that will raise rapidly in the gut lumen following microbiota installation. This process was previously reported in the mouse model by a mechanism that involves microRNA-146a-mediated translational repression and proteolytic degradation of the essential Toll-like receptor (TLR) signaling molecule interleukin-1 receptor-associated kinase 1 (IRAK1) [12]. This mechanism is sufficient to induce intestinal epithelial innate immune tolerance [13]. Although we did not demonstrate that similar mechanism occurs in lambs, we observed in this study that only in the gut loop explants, free of endotoxins, LPS induced chemokine production was preserved 5 h post-stimulation. Conversely, in explants generated with the ileum connected to the intestinal transit since 24 h, addition of LPS to these explants did not improve further chemokine upregulation with the exception of cxcl8 mRNA expression for which a slight increase was observed.

As a proof of concept, we next compared two YCW fractions for their immunostimulatory properties: one with a proportional content of β-glucans and mannoproteins and the second one enriched in β-glucans. We noticed that the YCW fractions enriched in β-glucans induced lower expression of pro-inflammatory cytokines in the gut-loop (CXCL8 and IL1α). Similar observation was previously made with macrophages cultured in vitro with *S. cerevisiae* extracts enriched in β-glucans that displayed weaker TLR2/4-related NFκB/AP-1 activity and less TNFα production [35]. We therefore can suspect that a similar mechanism may occur in gut lamb but this requires further investigations. When YCW1 and YCW2 were tested for their ability to reduce invasion and development of *C. parvum* in intestinal epithelial cells, they both limited in a modest but similar manner *C. parvum* early development despite different contents in β-glucans. One can conclude that the difference in ability to induce higher level of these immune effectors did not play a significant role in the protection process.

In addition to the evaluation of products for their immunostimulatory or their anti-infectious properties, the gut-loop in cesarean-born lambs can be used to further investigate host–microbial interactions in a controlled environment, and decipher immune feature of a specific area of the intestine. The gut-loop system is indeed suitable for investigations in various intestinal lymphoid and non-lymphoid segments by just making loops in the selected area as performed with jejunal loop made in a one-month-old piglet to study innate immune response to *Salmonella* [36]. Since our model does not require the use of antibiotics after the loop surgery, the neonatal gut-loop model can also be very useful to investigate the role of selected microbiota or probiotics that could be introduced in the “sterile loops” to evaluate their capacity to promote intestinal immune responses and mucosae maturation. For the later, maintenance of gut loop for long period would be required. A model of fetal lamb with a single 8–10 cm intestinal loop model was previously generated, and in this case, sterile intestinal loop constructed in utero retained functional GALT for as long as 6–7 months after birth [14]. This therefore demonstrates that even long-term slow progressive enteric diseases can be investigated with the gut-loop model. Our lamb gut-loop model could also be a model for human cryptosporidiosis to evaluate drug compounds and innate immune responses, considering the observed similarities between those of young ruminants and young children infected by *Cryptosporidium*. However, it has some limitations; for example, the large ileal Peyer’s patch of young ruminants, which is not present in human.

Overall, this model paves the way for further new control method development such as immunostimulants, antimicrobial compounds and vaccines dedicated to the control of enteric infections in neonates.

## Data Availability

The data presented in this study are openly available in Zenodo at [10.5281/zenodo.5235377].

## Supplementary Materials

The following are available online at https://www.mdpi.com/article/10.3390/vetsci8090170/s1, Table S1: List of forward and reverse primers used for gene expression analysis by RT-qPCR.
